# Bridging physiology and psychology: a new framework for teaching homeostasis

**DOI:** 10.3389/fphys.2026.1808472

**Published:** 2026-04-15

**Authors:** Suzan Kamel-ElSayed, Nermien E. Waly, Changiz Mohiyeddini

**Affiliations:** 1 Department of Foundational Medical Studies, Oakland University William Beaumont School of Medicine, Rochester Hills, MI, United States; 2 Medical Physiology Deparment, School of Medicine, Capital University (formerly Helwan), Helwan, Egypt

**Keywords:** allostasis, homeostasis, integration in medical education, medical education reform, medical physiology, mind-body interaction, psychology, psychophysiology

## Abstract

Classical physiology teaches homeostasis as a set of reactive feedback loops that stabilize the body’s internal environment. Yet emerging science shows that human regulation is not merely reactive but profoundly shaped by cognition, emotion, belief, and predictive brain processes. We propose an updated definition of homeostasis that positions the brain as an anticipatory, meaning-making regulator integrating physiological, emotional, and environmental demands. This predictive regulation also unfolds across time through circadian organization, in which central neural systems coordinate daily rhythms in endocrine, immune, metabolic, and cognitive activity to prepare the organism for anticipated environmental and behavioral demands. By reframing homeostasis as a psychophysiological process, this work highlights the need for medical education to move beyond reductionist feedback-loop models and adopt a more integrated, clinically relevant understanding of human regulation. Within this framework, cognitive expectations, emotional appraisal, and circadian timing together shape how the brain allocates physiological resources in anticipation of daily challenges. This framework offers a rigorous yet human-centered lens for interpreting health, disease, and the therapeutic power of the clinical encounter.

## 1 Introduction

Homeostasis is a cornerstone of physiology education, consistently defined in major medical textbooks as the dynamic regulation of the body’s internal environment to maintain key variables within a healthy range through coordinated physiological mechanisms. When conditions deviate from their set points, corrective processes are activated to restore balance.

The foundation of this concept was laid by Claude Bernard (1865), who established that the stability of conditions such as blood composition was essential for life. His work posited that internal constancy is a hallmark of healthy function. In the early 20th century, Walter Cannon (1929) formally defined homeostasis, shifting the focus from mere constancy to a regulated equilibrium maintained through dynamic feedback mechanisms. Modern textbooks, such as Guyton and Hall Textbook of Medical Physiology (2025) and *Medical Physiology* by Boron and Boulpaep (2021), further describe homeostasis as a dynamic, multilevel, and adaptive process operating from the molecular to the systemic level.

Despite these refinements, the prevailing pedagogical framework continues to teach homeostasis as a series of impersonal feedback loops, largely disembodied from the cognitive and emotional processes of the humans experiencing them. Grounded in reductionist animal experiments designed to isolate organ systems, this approach has inadvertently fostered a model of the body as a self-regulating machine. This diminishes the brain’s role to an “integrating switchboard” rather than the seat of a conscious, meaning-making self. Consequently, medical students are often underprepared to address the psychophysiological dynamics such as chronic stress exacerbating hypertension or anxiety disrupting gastrointestinal motility that they encounter in clinical practice.

## 2 Emerging paradigms of regulation: allostasis and predictive control

Cartesian dualism, proposed by René Descartes in the 17th century, posits that the mind and body are fundamentally separate substances. Emerging scientific paradigms are dissolving such a divide (Thibaut, 2018; Damasio, 1994). One framework is allostasis, which conceptualizes regulation as an anticipatory, brain-driven process rather than a passive, reflexive response to imbalance (Sterling and Eyer, 1988). Contemporary neuroscience and psychoneuroimmunology demonstrate that cognition, emotion, and physiological regulation are deeply integrated: the brain continuously coordinates cardiovascular, endocrine, and immune functions in response to internal and external demands. Allostasis posits that the brain functions as a predictive organ, continuously forecasting metabolic requirements and social demands to calibrate cardiovascular, immune, and endocrine responses. Within this framework, the brain is an active architect of physiological states, integrating learning, memory, and expectation into a cohesive regulatory strategy (Schulkin and Sterling, 2019). This process requires a sophisticated neural architecture capable of synthesizing internal interoceptive signals, environmental cues, and prior experience to orchestrate adaptive physiological shifts. The neural substrates of this mind-body interaction, specifically the prefrontal cortex, anterior cingulate cortex, insula, amygdala, and brainstem autonomic centers, constitute a central control network (Thayer and Lane, 2000; Critchley and Harrison, 2013). These regions coordinately regulate autonomic, endocrine, and immune activity while simultaneously mediating cognitive and emotional processing, enabling the generation of context-sensitive predictions. Furthermore, functional connectivity within this network is strongly correlated with heart rate variability (HRV) and stress resilience, illustrating how neural circuits implement predictive allostasis in real time. Ultimately, this framework demonstrates that the mind-body relationship is instantiated in identifiable neurobiological circuits, providing a rigorous mechanistic bridge between physiological regulation, cognition, and behavior.

### 2.1 Psychoneuroimmunology and neurovisceral integration

The field of psychoneuroimmunology (PNI) has established bidirectional communication pathways between the central nervous, endocrine, and immune systems. Chronic psychological stress is known to upregulate pro-inflammatory gene expression and accelerate cellular aging. Conversely, positive psychological states and mind-body interventions have demonstrated measurable beneficial effects on inflammatory markers and autonomic function. For instance, according to Huffman et al. (2023), research has identified connections between increased positive emotions and enhanced vagal tone, a key indicator of autonomic nervous system health. Furthermore, specific positive psychology interventions, such as optimism training for patients with heart disease, have led to significant improvements in health-related biomarkers, suggesting that cultivating positive affect can actively modulate the biological processes involved in medical prognosis.

In addition, neurovisceral integration research reveals how emotional regulation is directly reflected in autonomic balance, using HRV as a sensitive index of the brain’s capacity to modulate. Current literature highlights the Neurovisceral Integration Model, which proposes that the prefrontal cortex, the amygdala, and the autonomic nervous system form a unified system for self-regulation (Thayer and Lane, 2000). A key clinical implication of this model is the use of HRV as a non-invasive biomarker. High HRV is associated with superior executive function and emotional regulation, whereas low HRV is a risk factor for cardiovascular disease and inflammatory dysregulation. By integrating HRV monitoring into clinical practice, providers can move beyond “symptom management” to assess the patient’s actual capacity for physiological self-regulation and “inner peace” (Thayer et al., 2009).

PNI and neurovisceral integration represent complementary perspectives on the same integrated brain–body system. PNI has elucidated how psychological processes such as stress, emotion, and cognition influence immune and endocrine function, demonstrating that mental states are biologically instantiated rather than separable from physiology. Neurovisceral integration extends this insight by providing a mechanistic account of how higher-order brain networks regulate bodily systems through autonomic pathways, particularly via prefrontal–limbic control of cardiovascular and immune function. Together, these frameworks converge on a brain-centered, predictive model of regulation in which cognitive, emotional, and physiological processes are dynamically coordinated to support adaptive behavior. This integrated view aligns with allostatic principles, emphasizing anticipation, learning, and context-sensitive regulation rather than isolated, reactive responses.

### 2.2 Belief-driven regulation

Beliefs and expectations are not epiphenomenal to physiology; they are constitutive of it. Placebo and nocebo research demonstrates that expectations can activate endogenous opioid and dopaminergic systems, modulating physiological responses even in the absence of active compounds (Benedetti, 2022). Research on the “stress mindset” (Crum et al., 2017) further reveals that an individual’s interpretation of stress can shape their physiological response to challenges; specifically, viewing stress as enhancing rather than debilitating is associated with a more adaptive anabolic profile, characterized by a higher ratio of dehydroepiandrosterone (DHEA) to cortisol. This evidence suggests that subjective appraisal acts as a primary governor of the body’s internal milieu. Supporting this perspective, Huffman et al. (2023) highlighted those positive psychological traits such as optimism; essentially a belief-driven expectation of future success; are prospectively linked to superior prognostic biomarkers, including reduced pro-inflammatory cytokines (such as IL-6) and enhanced vagal tone. This supports the argument that the mind-body connection is a closed-loop system where the “software” of belief directly rewires the “hardware” of biological resilience. In a clinical context, this suggests that interventions targeting a patient’s belief systems (e.g., positive psychology interventions) are as biologically “real” as pharmacological treatments, as they utilize the same neurobiological pathways to modulate inflammation and cardiovascular health (Huffman et al., 2023).

### 2.3 Circadian rhythms

Circadian rhythms represent a fundamental temporal dimension of allostasis, organizing physiological and behavioral processes in anticipation of predictable environmental cycles (Rao and Androulakis, 2019; Sato, 2023). Governed by the suprachiasmatic nucleus (SCN), these rhythms coordinate hormone release, immune function, metabolism, and cognitive performance to align internal states with daily demands (Scheiermann et al., 2018). Unlike reactive homeostatic processes, circadian allostasis prepares the body in advance, minimizing the energetic cost of unexpected perturbations and reducing cumulative strain on physiological systems (Rao and Androulakis, 2019). Disruption of circadian timing through shift work, sleep deprivation, or misalignment with environmental cues can independently compromise metabolic, cardiovascular, and immune function, which in turn can exacerbate psychological stress, creating a self-reinforcing cycle of dysregulation (Sato, 2023). By framing circadian processes as predictive and integrative regulators, this framework highlights the brain’s central role in orchestrating temporal coordination across systems, offering a complementary perspective to other allostatic mechanisms such as belief-driven regulation.

### 2.4 Proposed psychophysiological framework

A reevaluation of core physiological tenets is mandated by this evidence. We propose a new, integrated definition:

“Homeostasis is the dynamic maintenance of the internal physiological environment through the integrated regulation of organ systems, driven by cognitive and emotional processes, anticipating internal and external demands to restore and sustain physiological balance and health.”

This definition moves away from viewing the mind as a passive observer and instead frames it as a core component of the body’s ability to sustain health.

## 3 Discussion

A central implication of the evidence reviewed above is that physiological regulation cannot be fully understood as a purely reactive process. Classical homeostasis describes the correction of deviations after they occur through negative feedback mechanisms. However, contemporary research in allostasis indicates that the brain continuously generates predictions about metabolic, environmental, and social demands and adjusts physiological activity in advance of disturbance. In this framework, regulation is not limited to restoring equilibrium but involves anticipatory coordination across autonomic, endocrine, and immune systems.

This anticipatory coordination also operates across predictable temporal cycles. Circadian rhythms represent a foundational example of feedforward physiological regulation in which the brain prepares bodily systems for expected daily demands before perturbations occur.

Repeated predictive activation has important consequences for health. When environmental or psychological conditions are persistently interpreted as threatening or demanding, regulatory systems are chronically engaged, producing what has been termed allostatic load, the cumulative physiological cost of sustained adaptive responses. This perspective helps explain why clinical outcomes are often poorly predicted by isolated physiological measurements alone. For example, hypertension may reflect not only vascular pathology but also repeated anticipatory activation of cardiovascular control systems driven by perceived stress and environmental context. Thus, treating symptoms without addressing the regulatory conditions that generate them may leave a primary driver of disease unmodified.

### 3.1 Expectation and belief as mechanisms of regulation

Within a predictive regulatory framework, cognition is not external to physiology but one of its mechanisms. Expectations represent the cognitive expression of prediction, shaping how the brain prepares the body for anticipated events. Mental stress provides a clear example of this brain–body integration. Experimental and clinical studies demonstrate that psychological stressors activate central autonomic networks that modulate cardiovascular, endocrine, and immune responses, linking emotional appraisal directly to measurable physiological outcomes (Carter and Goldstein, 2015). Importantly, cognitive expectations operate within the broader temporal architecture of circadian regulation. The same central neural systems that encode learned predictions about environmental and social demands interact with circadian networks in the hypothalamus to coordinate daily patterns of autonomic tone, endocrine secretion, immune activity, and cognitive performance. Findings from placebo and nocebo research demonstrate that beliefs and expectations can activate endogenous opioid, dopaminergic, and autonomic pathways, producing measurable physiological changes even in the absence of pharmacological agents. Similarly, studies on stress mindset show that how individuals interpret stress influences hormonal and autonomic responses, including cortisol dynamics and anabolic signaling.

These effects should not be viewed as exceptions to physiological regulation. Rather, they are observable examples of predictive control in humans. The brain uses prior experience, learning, and contextual interpretation to forecast demands and regulate bodily systems accordingly. From this perspective, patient expectations and clinician communication become biologically relevant variables capable of modulating inflammation, cardiovascular function, and recovery trajectories.

**Figure 1:**
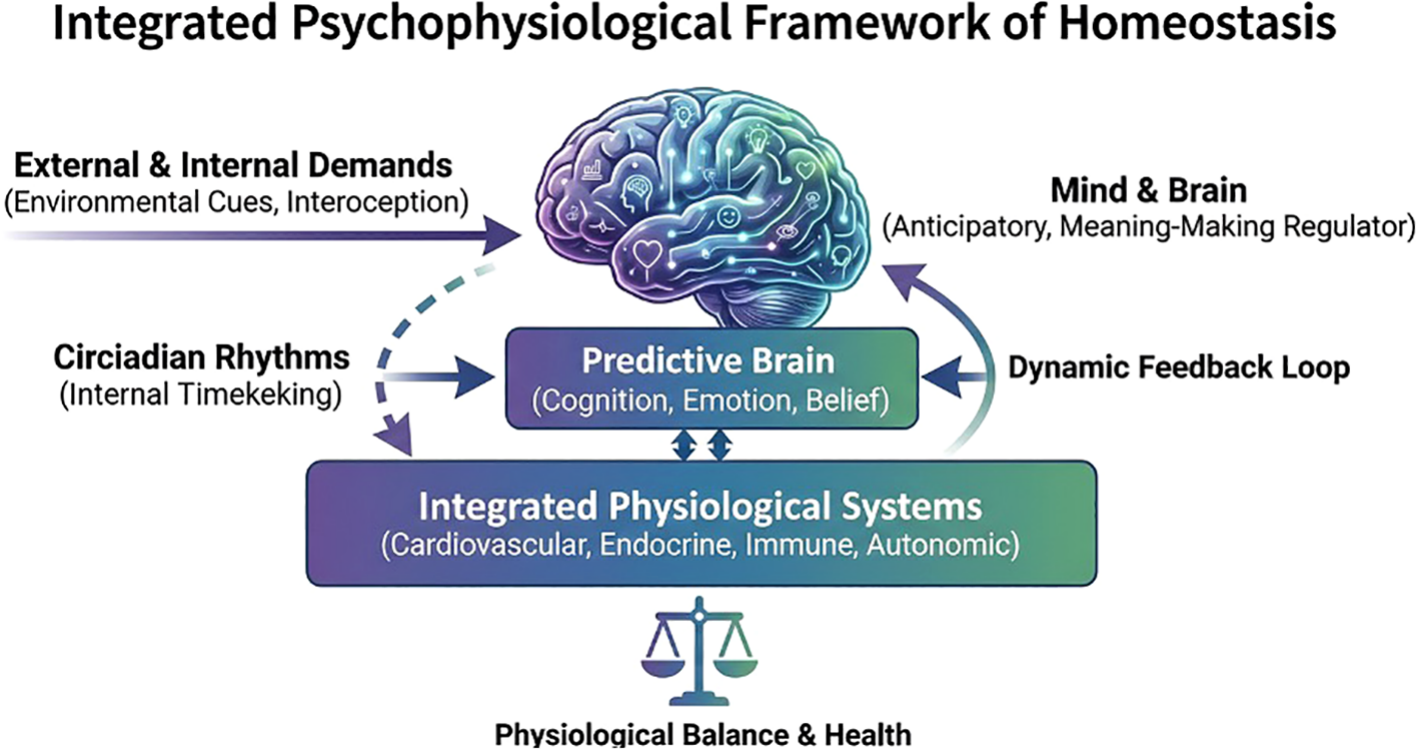
The integrated psychophysiological framework of predictive regulation (AI generated). A schematic representation of the proposed definition of homeostasis, illustrating the shift from a reactive feedback loop to a predictive, anticipatory model where the brain integrates environmental cues, interoception, circadian rhythms and belief systems to regulate physiological balance.

**Figure 1** summarizes this proposed model: environmental context, interoceptive input, and learned expectations are integrated within central neural networks, which in turn regulate autonomic, endocrine, and immune activity to maintain functional stability. The clinical encounter therefore participates in regulation, not merely in its observation.

### 3.2 The shift from reactive to predictive regulation

A major advance in contemporary physiology is the transition from a purely reactive model of homeostasis to the predictive model of allostasis. While classical homeostasis focuses on correcting deviations after they occur (feedback), allostasis emphasizes the brain’s ability to anticipate metabolic needs based on environmental and psychological cues (feedforward) (Sterling, 2012). Circadian rhythms provide one of the clearest biological manifestations of this anticipatory architecture. By synchronizing physiological systems with the light–dark cycle and expected behavioral patterns such as feeding, activity, and sleep, circadian networks reduce the metabolic cost of reactive responses and help maintain regulatory efficiency across the day.

Clinically, this explains why “allostatic load”, the physiological wear and tear resulting from chronic overactivity or underactivity of stress-responsive systems, is a better predictor of long-term health outcomes than isolated homeostatic markers (McEwen, 1998). For the clinician, treating a symptom (e.g., hypertension) without addressing the chronic psychological “anticipatory stress” that drives it is an incomplete intervention.

### 3.3 Mindset as a clinical variable

The “stress mindset” research by Crum et al. (2017) provides a transformative perspective on patient education. Their studies demonstrate that shifting a patient’s belief from “stress is debilitating” to “stress is enhancing” significantly alters their cortisol response and increases growth-oriented hormones like DHEA. Similarly, the neurobiology of the placebo and nocebo effects (Benedetti, 2014) proves that a clinician’s communication style and a patient’s expectations can activate endogenous opioid and dopaminergic pathways. These are not merely “subjective” changes; they are objective biochemical modulations that can accelerate recovery or exacerbate disease.

### 3.4 Pedagogical approaches: teaching the new definition of homeostasis

A revised definition of homeostasis grounded in predictive, brain-centered regulation carries important implications for medical education. Traditional organ-system–based curricula often segregate physiology and psychology, limiting learners’ ability to conceptualize health as an integrated, adaptive process. Teaching homeostasis through an allostatic lens requires pedagogical approaches that emphasize cross-system coordination, anticipatory control, and context sensitivity rather than static set-point correction. Educational strategies that foreground trans-systemic pathways; linking emotional appraisal, autonomic regulation, endocrine signaling, and immune function; can help learners recognize how regulatory processes unfold across neural and bodily systems. Case-based and team-based learning formats provide natural opportunities to examine how patients’ interpretations of illness modulate hypothalamic–pituitary–adrenal axis activity and downstream inflammatory responses in clinically relevant contexts such as perioperative recovery or chronic disease. Core regulatory constructs, including allostasis and autonomic flexibility, can be integrated alongside traditional pathophysiology to reframe disease as the cumulative consequence of sustained predictive demand. Physiological markers such as HRV offer a concrete means of operationalizing regulatory capacity, linking cortical control mechanisms to measurable indicators of resilience and cardiovascular risk. For instance, a TBL application exercise might ask teams to predict how a patient’s catastrophizing cognitions about chest pain would alter HRV and inflammatory markers during emergency department evaluation, then trace the neural pathways mediating these effects. Experiential and simulation-based approaches further reinforce these principles by making neural and autonomic dynamics observable in real time, while attention to the clinical encounter itself highlights communication as a biologically instantiated process capable of shaping patients’ physiological states. Finally, emerging models proposed by Huffman et al. (2023), in medical populations underscore the feasibility of incorporating low-burden, psychologically informed interventions into routine care, illustrating how influencing the brain’s predictive architecture can yield durable improvements in health behaviors and prognostic biomarkers.

### 3.5 Critical evaluation and future directions

The proposed framework does not suggest that all physiological variables are equally influenced by cognitive processes. Regulatory systems differ in the degree to which they are accessible to predictive modulation. Parameters maintained within narrow survival ranges, such as blood pH or plasma osmolarity, are tightly constrained by local biochemical and reflexive mechanisms. In contrast, systems involved in adaptation to environmental and behavioral demands—including autonomic balance, inflammatory activity, metabolic regulation, and endocrine stress responses—are more plausibly shaped by learning, context, and appraisal.

Current evidence most strongly supports modulation of systemic physiological states (e.g., autonomic tone, inflammatory signaling, and neuroendocrine activity) rather than direct alteration of fixed set-points. The framework therefore should be understood as extending, not replacing, classical homeostasis. Feedback stabilization remains essential, but it operates alongside predictive coordination implemented by central neural networks.

Future work should also clarify how cognitive appraisal and belief interact with circadian regulatory systems, as temporal misalignment (e.g., sleep disruption or shift work) may amplify the physiological impact of psychological stress and contribute to cumulative allostatic load.

Effect sizes of psychologically informed interventions are often modest, and individual variability is substantial. Genetic differences, developmental history, and interoceptive sensitivity likely influence responsiveness to predictive regulatory processes. Early life experiences shape the brain’s predictive architecture by calibrating neural systems involved in threat detection, stress appraisal, and interoceptive interpretation. Research on adverse childhood experiences (ACEs) and early trauma demonstrates robust associations with long-term alterations in autonomic regulation, hypothalamic–pituitary–adrenal axis function, inflammatory signaling, and cardiometabolic risk. This developmental perspective further reinforces the importance of integrating psychological and physiological processes in medical education and clinical care. Patients’ regulatory patterns cannot be fully understood without considering the experiential histories that shape how their brains interpret and anticipate environmental demands.

Future research should employ longitudinal and experimental human studies to determine which physiological domains are most sensitive to cognitive and contextual modulation and to clarify the mechanisms linking predictive brain activity with measurable clinical outcomes.

## 4 Conclusion

The next evolution in our understanding of homeostasis must bridge mechanistic physiology with the psychophysiological realities of human health. By viewing humans as embodied beings, we provide a framework that is both mechanistically accurate and holistically human. This aligns with the NIH’s “whole person health” framework, which emphasizes that health and disease emerge from dynamic interactions among biological, behavioral, psychological, social, and environmental factors rather than isolated organ dysfunction (https://www.nccih.nih.gov/health/whole-person-health-what-it-is-and-why-its-important) (National Center for Complementary and Integrative Health, 2021).

The proposed perspective also resonates with the foundational tenets of osteopathic medicine, which emphasize the unity of the body, mind, and spirit and the body’s inherent capacity for self-regulation and healing (https://osteopathic.org/about/leadership/aoa-governance-documents/tenets-of-osteopathic-medicine/) (American Osteopathic Association, n.d.).

## Data Availability

This manuscript did not involve the collection, generation, or analysis of any data, code, or other materials from external sources, and thus no data statement is applicable.
